# Acute Clinical Syndromes and Suspicion of SARS-CoV-2 Infection: The Experience of a Single Romanian Center in the Early Pandemic Period

**DOI:** 10.3390/medicina57020121

**Published:** 2021-01-29

**Authors:** Raluca Ecaterina Haliga, Victorita Sorodoc, Catalina Lionte, Ovidiu Rusalim Petris, Cristina Bologa, Adorata Elena Coman, Luminita Gina Vata, Gabriela Puha, Gabriela Dumitrescu, Oana Sirbu, Alexandra Stoica, Alexandr Ceasovschih, Mihai Constantin, Andreea Nicoleta Catana, Elisabeta Jaba, Laurentiu Sorodoc

**Affiliations:** 1 Department of Internal Medicine, “St. Spiridon” Emergency Clinical Hospital, 700111 Iași, Romania; victorita.sorodoc@umfiasi.ro (V.S.); catalina.lionte@umfiasi.ro (C.L.); ovidiu.petris@umfiasi.ro (O.R.P.); cristina.bologa@umfiasi.ro (C.B.); ado_coman@yahoo.com (A.E.C.); luminita.vata@umfiasi.ro (L.G.V.); gabriela.puha@umfiasi.ro (G.P.); dumitrescu.gabriela@umfiasi.ro (G.D.); oana.sirbu@umfiasi.ro (O.S.); alexandra.rotariu@umfiasi.ro (A.S.); alexandr.ceasovschih@umfiasi.ro (A.C.); mihai.s.constantin@umfiasi.ro (M.C.); laurentiu.sorodoc@umfiasi.ro (L.S.); 2 Faculty of Medicine, “Gr. T. Popa” University of Medicine and Pharmacy, 700115 Iași, Romania; 3 Department of Infectious Diseases, “St. Spiridon” Emergency Clinical Hospital, 700111 Iași, Romania; catana.andreea87@gmail.com; 4 Statistics Department, FEEA, “Al. I. Cuza” University, 700506 Iasi, Romania; elisjaba@gmail.com

**Keywords:** acute clinical syndromes, suspicion of SARS-CoV-2 infection, differential diagnosis

## Abstract

*Background and Objectives*: During the coronavirus disease 2019 (COVID-19) pandemic, patients with chronic diseases suffering exacerbations have required acute medical care. The purpose of our study was to determine useful criteria for the differentiation of patients with acute clinical syndromes and suspicion of severe acute respiratory syndrome coronavirus 2 (SARS-CoV-2) infection. *Materials and Methods*: This was an observational retrospective study, conducted in an internal medicine clinic from April to May 2020. We collected clinical, biological, and computed tomography (CT) data on patients with exacerbations of chronic diseases and clinical suspicion of SARS-CoV-2 infection. Patients with an already-positive real-time reverse-transcription polymerase chain reaction (RT-PCR) test for SARS-CoV-2 on presentation at the emergency department were excluded from our study. *Results*: Of 253 suspected cases, 20 were laboratory-confirmed as having SARS-CoV-2 infection by RT-PCR, whereas COVID-19 diagnosis was ruled out in the remaining 233. Venous thromboembolism (VTE) correlated significantly with COVID-19 diagnosis in suspected patients, while laboratory markers were not significantly different between the two groups. Of the suspected patients, significantly higher percentages of dry cough, fever, myalgias, sore throat, loss of smell and appetite, and ground-glass opacities (GGOs) on CT were found in SARS-CoV-2-positive individuals. *Conclusions*: The study demonstrated that, until receiving the result of an RT-PCR test for SARS-CoV-2 (usually 12–24 h), association with VTE as a comorbidity, fever, dry cough, and myalgia as clinical features, and GGO on CT are the main markers for the identification of COVID-19 patients among those suspected with acute clinical syndromes. Our results also provide evidence for doctors not to rely solely on biological markers in the case of suspected SARS-CoV-2 infection in patients with exacerbations of chronic diseases. These data are useful for faster decision-making with regard to suspected COVID-19 patients before receiving RT-PCR test results, thus avoiding keeping patients in crowded emergency departments.

## 1. Introduction

In December 2019, the novel severe acute respiratory syndrome coronavirus 2 (SARS-CoV-2) was identified in relation to a newly emerging type of viral pneumonia, which was subsequently named coronavirus disease 2019 (COVID-19). In March 2020, COVID-19 was declared a worldwide pandemic [1,2]. To date, more than 99 million cases of SARS-CoV-2 infection have been detected around the world, with over 2 million deaths reported due to this disease [3]. Thousands of articles have reported the characteristics and changes in COVID-19 patients with respect to clinical, biological, or radiological features, as well as treatment options related to guidelines [1,2,4,5,6,7], with the majority of these studies focused strictly on the disease and its complications. During the pandemic, patients with chronic diseases suffering exacerbations have needed acute medical care. Unfortunately, it is also a reality that the clinical signs and symptoms and the biological or radiological changes of COVID-19 can overlap with those of other acute diseases or the exacerbations of chronic ones. To our knowledge, there are few studies related to the differentiation of confirmed COVID-19 from suspected but confirmed SARS-CoV-2-negative patients, as the differentiation of suspected SARS-CoV-2 infection and acute diseases is difficult in terms of clinical, biological, and/or radiological criteria [8,9].

In this study, we included patients with exacerbations of chronic diseases (cardiac, respiratory, metabolic, and digestive diseases or their combinations) and clinical suspicion of SARS-CoV-2 infection. The aim of the study was to analyze whether the evaluation of clinical symptoms and signs, blood tests, and thoracic computed tomography (CT) scanning upon admission to hospital can be useful for the differentiation of patients with acute medical pathology and suspicion of SARS-CoV-2 infection. The results of this study show the significance, as well as the sensitivity and specificity, of these objective markers for diagnosis of COVID-19 in patients with acute medical illnesses, thus enabling physicians to differentiate these situations more effectively and adjust their management accordingly.

## 2. Materials and Methods

This was an observational, retrospective study performed on a cohort of patients admitted to our internal medicine clinic at the emergency clinical county hospital in a large city in the northeast region of Romania over two months. Thus, between 1 April and 31 May 2020 we were mainly responsible for the admission and differential diagnosis of patients suspected to have COVID-19 with acute medical illnesses. Improving diagnostic efficiency became mandatory and essential for differentiating between the two situations (COVID-19 vs. non-COVID-19).

During the period of study (April–May 2020), there were on average of 300 cases of COVID-19/day in Romania confirmed from approximately 15,000 RT-PCR tests for SARS-CoV-2/day (the Romanian population in 2020 was 19.4 million people). The period of lockdown also reduced the population’s access to medical care, whereby only urgent cases presented to hospitals and emergency departments.

We enrolled patients who were over 18 years of age, irrespective of gender, that were admitted to the emergency department (ED) due to clinical manifestations of acute medical illnesses as a consequence of exacerbation of their chronic diseases, as well as symptoms or clinical signs included in the case definition of suspected cases of SARS-CoV-2 infection (Table 1).

All patients (or their families in the case of unconscious subjects) signed an informed consent form prior to admission to our hospital. No experimental research was carried out on any patient. Patients without a signed informed consent form, those aged younger than 18 years, those with an acute pathology which needed specific emergency treatment (i.e., surgery, trauma, burns, acute stroke, etc.), or with a RT-PCR test already positive for SARS-CoV-2 at presentation to the ED were excluded from our study.

All patients underwent routine evaluation and management in the ED, which involved clinical examination, measurement of blood parameters (complete blood count), biochemistry profiling (glucose, renal and liver function tests, and electrolytes), arterial blood gas analysis, and an electrocardiogram (ECG). Measures of basic or advanced cardiac life support were applied for patients with severe acute illness. The patients were then admitted to our clinic or to the intensive care unit (ICU) department. 

All patients admitted to our clinic underwent RT-PCR testing for SARS-CoV-2. Moreover, venous blood samples were collected for other specific tests (C-reactive protein (CRP), D-dimer, ferritin, lactate dehydrogenase (LDH), creatine kinase MB (CKMB), and troponin). Some hospitalized patients also underwent chest CT depending on the indication for this investigation [7].

The patients were observed only during hospitalization. Our main purpose was the treatment and stabilization of their acute illnesses. A favorable outcome was considered with regard to patients who were discharged with improved symptoms, while a poor outcome was represented by in-hospital death. All patients who received a positive RT-PCR result for SARS-CoV-2 were transferred to an infectious disease hospital for specialized treatment of COVID-19.

Statistical analyses were performed with SPSS software for Windows (v.22.0; SPSS, Chicago, IL, USA). Nominal variables are presented as frequencies and percentages, and continuous variables are presented as the mean ± standard deviation (SD). To identify significant parameters associated with COVID-19 diagnosis, a two-tailed Student’s *t*-test was used to compare normally distributed continuous variables, whereas the chi-squared test and Cochrane’s statistic were used for categorical variables. All significant variables in the univariate analyses for diagnosis were subjected to a multivariate logistic regression analysis. Risk was expressed as relative risk (RR) or odds ratio (OR) with confidence intervals (CIs). Optimal cutoff points for the parameters analyzed were determined using the area under the receiver operating characteristic (ROC) curve (AUC) with 95% CI and the associated *p*-value representing the likelihood of the null hypothesis (AUC = 0.5). A *p*-value < 0.05 was considered statistically significant.

## 3. Results

The study included 253 patients (57.7% men), with a mean age of 63.66 years (range 19–99 years). Depending on the result of the RT-PCR test for SARS-CoV-2, the patients were divided into two groups: those with a positive test (the SARS-CoV-2 POS group) and those with a negative test (the SARS-CoV-2 NEG group). Thus, from a total of 253 patients, 7.9% (20 patients) were SARS-CoV-2 POS and 92.1% (233 patients) were SARS-CoV-2 NEG. The patients’ chronic treatment was either continued or adapted for the particularities of acute medical illnesses; however, this did not influence the results of the study.

Of the 253 patients included in the study, 236 patients were admitted to the clinic and 17 patients were admitted to the ICU department. None of the patients with confirmed SARS-CoV-2 according to RT-PCR (20 patients) were admitted to the ICU; they were instead transferred to an infectious disease hospital.

Regarding the demographic characteristics (age, gender, and residence) and smoking habits of the patients, there was no significant association found with regard to a positive result for SARS-Cov-2 test (*p* > 0.05). A higher percentage (45.0%) of SARS-CoV-2 POS patients were between 40 and 59 years old, while 43.8% of SARS-CoV-2 NEG patients were older (60–79 years old).

### 3.1. Chronic Underlying Diseasesin Patients Suspected to Have COVID-19

The distribution of selected chronic diseases among patients included in the study (obesity, diabetes mellitus, hypertension, chronic coronary syndrome, chronic heart failure, atrial fibrillation, venous thromboembolism (VTE), chronic obstructive pulmonary disease (COPD), pulmonary fibrosis, liver cirrhosis, and chronic kidney disease) and the estimated risks of COVID-19 diagnosis are included in Table 2. Patients with a positive RT-PCR SARS-CoV-2 test had >3-fold higher relative risk of venous thromboembolism (RR = 3.26; 95% CI: 1.09–9.72; *p* = 0.037). The estimated risk of a positive RT-PCR SARS-CoV-2 test was also higher in obese patients, but without statistical significance (RR = 1.17; 95% CI: 0.45–3.09; *p* = 0.751). The percentages of patients with chronic heart failure, atrial fibrillation, or COPD were significantly higher in the SARS-CoV-2 NEG group (Table 2). There were also two patients (10.0%) with acute coronary syndrome in the SARS-CoV-2 POS group (one with unstable angina and one with ST-elevation myocardial infarction (STEMI)), as compared with 11 patients (4.72%) in the SARS-CoV-2 NEG group (seven without STEMI and four with STEMI). In addition to the pathologies included in Table 2, patients presented other respiratory or digestive diseases. There were seven patients with bronchial asthma, with only one (5.0%) in the SARS-CoV-2 POS group; 21 patients with bronchiectasis, with 20 (8.6%) in the SARS-CoV-2 NEG group; and three patients with pulmonary cancer, with all in the SARS-CoV-2 NEG group. In terms of digestive diseases, 39 patients had chronic hepatitis, with the majority (36, 92.3%) in the SARS-CoV-2 NEG group, whereas three patients had inflammatory bowel disease, 10 patients had peptic ulcers, and 12 patients had digestive cancer, with all in the SARS-CoV-2 NEG group; however, the differences were not statistically significant between the two groups (Table 2).

### 3.2. Clinical Symptoms and Signs in Patients Suspected of Having COVID-19

SARS-CoV-2 POS patients had a significantly higher percentage of a documented history of fever at home and dry cough (60% for both) than SARS-CoV-2 NEG patients (31.8% and 33.9%, respectively) (*p* < 0.05) (Table 3), as well as significantly higher percentages of myalgia, sore throat, loss of smell, and loss of appetite (Table 3). The percentage of patients with dyspnea was significantly higher in SARS-CoV-2 NEG patients, in concordance with the higher percentage of patients with chronic heart failure in this group. Symptoms such as asthenia, headache, cough with sputum production, chest pain, arthralgia, dysphonia, dysgeusia, nausea, vomiting, abdominal pain, and diarrhea were not significantly different between the two groups *(p* > 0.05) (Table 3).

Of the 233 patients in the SARS-CoV-2 NEG group, 207 patients (81.8%) were discharged with improved symptoms after treatment, while 26 patients died (10.3%) as a result of complications and the evolution of their acute medical illness. A total of 17 (6.7%) SARS-CoV-2 NEG patients were admitted to the ICU for life support measures (Table 4). There were 20 patients (7.9%) with a positive RT-PCR result for SARS-CoV-2, all of whom were transferred to an infectious disease hospital for specialized treatment of COVID-19 (Table 4).

### 3.3. Biological Changes in Patients Suspected to Have COVID-19

Immediately after admission to the clinic and before receiving RT-PCR results for SARS-CoV-2 (usually 12–24 h), patients were evaluated with specific blood tests (CRP, D-dimer, ferritin, LDH, CKMB, and troponin) and chest CT, when indicated. None of the biological tests presented significant differences between the SARS-CoV-2 POS and SARS-CoV-2 NEG groups, highlighting once again that all these patients had acute exacerbations of chronic diseases (Table 5).

### 3.4. CT Changes in Patients Suspected of Having COVID-19

**Figure 1:**
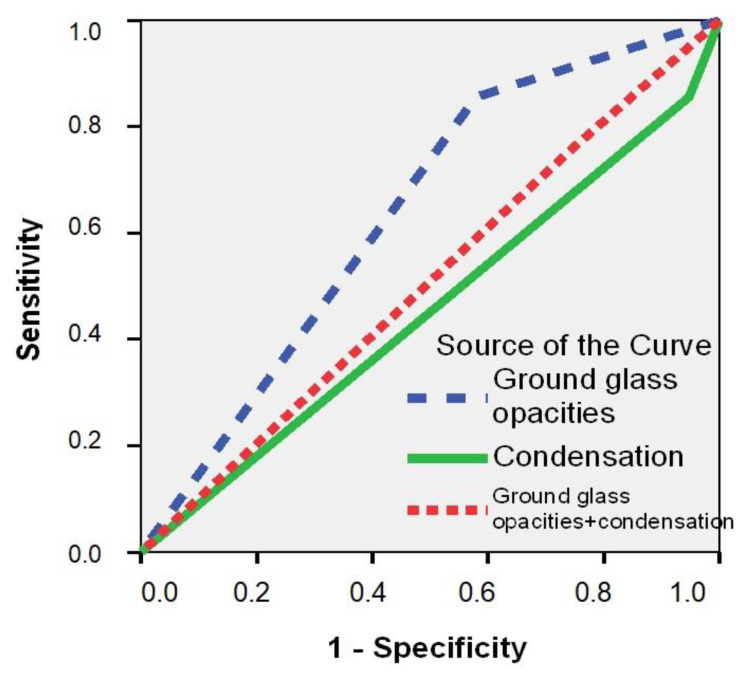
Receiver operating characteristic (ROC) curve for prediction of a positive RT-PCR result for SARS-CoV-2 as a function of CT changes.

In total, nine (45.0%) SARS-CoV-2 POS patients had ground-glass opacity (GGO) on their CT scan, which was a significantly higher percentage compared to the 14.6% (34) of SARS-CoV-2 NEG patients (*p* = 0.002) (Table 6). Patients with a positive SARS-CoV-2 RT-PCR test had an almost four-fold increased estimated risk of GGO on their CT scan (RR = 3.99; 95% CI: 1.76–12.4; *p* = 0.002). The ROC curve confirmed that the GGO aspect of CT is a good predictor for a positive RT-PCR SARS-CoV-2 result (AUC = 0.652; 95% CI: 0.513–0.791; *p* = 0.024) (Figure 1). The appearance of consolidation on CT and the association with GGO, along with statistical significance, are described in Table 6 (*p* ˃ 0.05).

**Figure 2:**
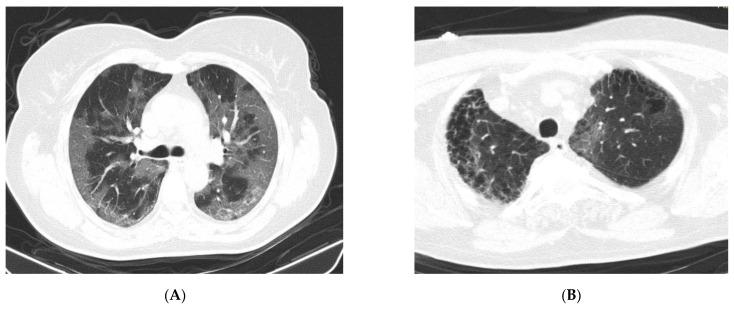
Native chest CT. (**A**) Images of extensive ground-glass opacity (GGO), distributed bilaterally, centrally, and peripherally. (**B**) GGO image in a left apical subpleural location (interstitial lesion in pulmonary fibrosis, with emphysema bubbles), suggestive of COVID-19 pneumonia (negative RT-PCR test) (from the collection of the radiology clinic, St. Spirid on Hospital, Iasi, Romania, May 2020).

Regarding CT changes in the SARS-CoV-2 POS group, four patients (20.0%) showed a nontypical COVID-19 distribution. One patient (5.0%) presented a unique central GGO image and three patients (15.0%) had multiple bilateral central GGO images. In five patients (25.0%), typical CT features were described, with multifocal bilateral GGO distribution (e.g., Figure 2A). A total of six COVID-19 patients (30.0%) showed CT findings of confluent GGO and bilateral consolidation, which are characteristic of severe forms of SARS-CoV-2 infection. Five COVID-19 patients had normal CT scans.

In our study, there were 11 cases (4.3%) with a typical CT appearance of COVID-19 pneumonia, along with laboratory indications of inflammatory alterations, but with a negative RT-PCR test for SARS-CoV-2. Serological testing for COVID-19 was not performed; however, in these patients, a repeat RT-PCR test for SARS-CoV-2 after 6–7 days remained negative. There were also patients in the SARS-CoV-2 NEG group for whom the CT features were suggestive of COVID-19 (e.g., Figure 2B).

Univariate logistic regression analysis showed that several variables were significantly and independently correlated with a positive SARS-CoV-2 RT-PCR result; however, only VTE in terms of personal history, myalgia and loss of appetite in terms of clinical symptoms, and GGO in terms of CT aspects showed a predictive value for COVID-19 diagnosis in patients with acute clinical syndromes after multivariate logistic regression analysis (Table 7). History of fever at home, dry cough, and myalgia, from a clinical point of view, were variables with a higher sensitivity for a positive SARS-CoV-2RT-PCR test in patients with acute medical illnesses (˃65%), while the specificity was >45% (Table 8). The CT aspect of GGO had a sensitivity for COVID-19 diagnosis of 85%, while specificity was 45% (Table 6). Both clinical (history of fever at home, dry cough, and myalgia) and CT (GGO) aspects were demonstrated to be good predictors for COVID-19 diagnosis using ROC methodology (AUC ˃ 0.600) (Table 8).

## 4. Discussion

Patients with chronic diseases can suffer exacerbations of their pathologies, and their symptoms can overlap with the clinical signs or symptoms of COVID-19 patients. To our knowledge, there are few studies related to the differentiation, diagnosis, and management of patients with exacerbations of chronic diseases and clinical suspicion of SARS-CoV-2 infection [8,9].

Our study was performed over two months in the early pandemic period (April–May 2020), when Romania was in complete lockdown. During that period, our clinic was mainly responsible for the admission and differential diagnosis of patients with acute illnesses and symptoms suggestive of COVID-19 who were admitted to our hospital; the patients with confirmed SARS-CoV-2 infection by RT-PCR tests were transferred to an infectious disease hospital for specialized treatment. Patients already confirmed as having SARS-CoV-2 infection on presentation at emergency department were not included in our study. In the early pandemic period, Romania had an average of 300 confirmed cases of COVID-19/day, diagnosed from approximately 15,000 RT-PCR tests for SARS-CoV-2/day. Lockdown reduced the population’s access to medical care, whereby only urgent cases were seen in hospitals and emergency departments. During that period, the infectious diseases hospital in our region admitted only patients with RT-PCR-confirmed SARS-CoV-2 infections (with approximately 350 beds in the hospital). At that time, our clinic was not responsible for COVID-19 patient monitoring and treatment, but only for diagnosis. For these reasons, the number of COVID-19-positive patients in the present study is small (*n* = 20).

Our results showed that patients with appositive SARS-CoV-2 RT-PCR test did not have a higher mean age, with the difference between the two groups being nonsignificant. In some previous studies, patients diagnosed with COVID-19 were found to be younger (mean age 45 years old) [10,11], while in others, the confirmed patients were older than the excluded patients [8]. However, COVID-19 can occur in patients of all age groups and does not have a typical age of onset [12]. One possible explanation is that, in our study, the patients were older than in other studies (mean age 63.66 ± 15.87 years old) as they had one or more chronic diseases. In the SARS-CoV-2 POS group, nonsmokers represented a higher percentage compared to the SARS-CoV-2 NEG group, although the difference was nonsignificant. Smoking is another controversial issue related to COVID-19, with some studies showing an association of smoking status with severity, negative progression, and adverse outcomes of SARS-CoV-2 infection [13], while other studies have not reported smoking as a risk factor for disease appearance and evolution [14].

SARS-CoV-2 POS patients had a significantly higher relative risk of VTE in our study, with this risk being higher for obese patients. However, there was a nonsignificant difference compared to the SARS-CoV-2 NEG group. These results are in concordance with other studies, which sustained relationships between SARS-CoV-2 infection, coagulation disorders, and obesity. SARS-CoV-2 is implicated in the pathogenesis of thromboembolic events indifferent mechanisms [15]; the rate of VTE is reportedly different depending on the severity of COVID-19, with a higher incidence in severe ICU patients [16,17,18]. Obesity-induced adipose tissue inflammation and its effects on the immune system also play a crucial role in the pathogenesis of COVID-19 infection [19]; however, obese patients are at a higher risk of severe evolution of COVID-19, while our patients suspected as being positive did not have severe forms of SARS-CoV-2 infection.

All suspected patients included in our study had clinical signs and symptoms in the context of exacerbation of their chronic underlying diseases, with the most significant ones for SARS-CoV2 POS patients being history of fever at home, dry cough, myalgia, sore throat, loss of smell, and loss of appetite, data which are in accordance with previously published studies [6,10,20,21,22]. Fever and dry cough were the most frequently observed symptoms in patients suspected of being positive. The percentage of patients with dyspnea was significantly higher in patients suspected of being negative, which was related to a higher percentage of patients with chronic heart failure in this group.

Laboratory findings were not significantly different between the two groups. An increase in inflammatory markers (CRP) and reduced lymphocyte count occurred datal most identical rates in patients suspected of being positive and negative (see Table 5). Levels of D-dimer were increased in a higher proportion of negative patients, while increases in LDH, CKMB, and troponin occurred at similar rates in both groups. This situation can be explained by all patients having acute clinical syndromes, giving rise to different changes in laboratory measures. Some patients had chronic coronary syndromes and acute coronary syndromes (in which LDH, CKMB, troponin, and CRP levels increased), chronic heart failure and acute heart failure (with an increase in cardiac enzymes as a result of myocardial injury [23]), VTE (with an increase in D-dimer levels as well as cardiac enzymes [24]), acute inflammatory conditions such as exacerbations of COPD or septic conditions (with an increase in CRP, as well as D-dimer or ferritin [25,26,27,28]), and chronic liver disease (in which LDH, ferritin [29], and D-dimer levels can increase [30]).

Other studies compared the laboratory data of patients with different levels of COVID-19 severity, finding that LDH, CKMB, cardiac troponin I, and/or D-dimer levels were significantly higher in severely critically ill patients due to infection of the myocardium by SARS-CoV-2 followed by myocardial injury [1]. Moreover, CRP levels were found to be higher [22], with a decrease in lymphocyte count in severe forms of COVID-19 [10]. These findings were not present in our study as patients were severely ill due to the exacerbation of chronic diseases.

A high frequency of ground-glass opacities (GGOs) was observed in the chest CT scans of patients suspected of being positive in our study, with a significant increase compared to those suspected of being negative. These results are consistent with previous studies, with typical CT findings in individuals with COVID-19 involving GGOs [22]. GGO patterns are significantly prevalent in the early phase of the disease [5], consistent with our study where patients suspected of being positive were in an early phase of COVID-19. In studies on suspected patients, a very high proportion of confirmed patients (83.3%) presented GGOs, whereas other CT aspects such as consolidation or pleural effusion were described but were not significantly different [8]. Other authors reported that pure/mixed GGO scans can also be common in negative patients, because many pulmonary infectious diseases (such as influenza virus pneumonia) and noninfectious conditions (including interstitial pulmonary edema, pulmonary hemorrhage, organizing pneumonia, or alveolar proteinosis) can show similar appearances on imaging [9,30]. A higher but nonsignificant percentage of consolidation was found in patients suspected of being negative in our study, with pneumonia in these cases having other etiologies.

According to the Radiological Society of North America (RSNA) expert consensus, the most commonly reported and typical imaging features with greater specificity for COVID-19 pneumonia are peripheral, bilateral, or multifocal GGO, with or without consolidation. Aspects of indeterminate appearance, described as nonspecific imaging features of COVID-19 pneumonia, include the absence of typical features and the presence of multifocal, diffuse, perihilar, or unilateral GGO with or without consolidation, lacking a specific distribution. Uncommonly or nonreported features of COVID-19 pneumonia include the absence of typical or indeterminate features and the presence of isolated lobar or segmental consolidation without GGO, discrete small nodules, lung cavitation, or smooth interlobular septal thickening with pleural effusion [31].

This study showed that, in terms of underlying comorbidities, only VTE was significantly correlated with COVID-19 diagnosis in suspected patients. History of fever at home, dry cough, and myalgia (in terms of clinical symptoms) and GGO (in terms of CT appearance) were demonstrated to be good predictors for COVID-19 diagnosis in suspected patients using ROC methodology. Furthermore, the sensitivity and specificity of GGO for COVID-19 diagnoses were high (85% and 45%, respectively). Similar results were found in other studies, with a high sensitivity of CT in SARS-CoV-2-positive patients (86–97%) [22,32].

Our study has several limitations. First of all, it was a single-center study, performed in an Internal Medicine clinic at an emergency clinical county hospital in the Moldavian region of Romania. Secondly, the study had a relatively small number of patients, observed over a short period of time, and thus it may not fully reflect the characteristics of all suspected patients. The data was collected during the early pandemic period, when Romania had a small number of confirmed cases of COVID-19 per day, while the number of RT-PCR tests for SARS-CoV-2 per day was not high enough. Thirdly, during the pandemic period, some investigations (e.g., bronchoscopy) were not carried out due to a biosafety risk. Lastly, we are aware that the results of our study, related to the presence of comorbidities as well as clinical and CT aspects, are known criteria for COVID-19 patients, and the current understanding and investigation methods has advanced rapidly since the time of the study. Current guidelines also include SARS-CoV-2 rapid antigen test for diagnosis, but the sensitivity and specificity of the antigen assay is inferior to those of the RT-PCR assay. We rely mainly on the PCR-based test, which takes longer (12–24 h), when patients are in need of medical care. Thus, this study is mainly aimed at internists practicing in emergency hospitals who are responsible for the admission of patients with acute exacerbations of chronic diseases and symptoms suggestive of COVID-19; the aim was to improve diagnostic efficiency and increase the possibility of a rapid differential diagnosis between suspected and confirmed cases as a function of a higher frequency of VTE, symptoms such as fever, dry cough, or myalgia, and CT findings such as GGO, but not as a function of any biological markers.

## 5. Conclusions

The admission and differential diagnosis of patients with acute exacerbations of chronic diseases (a very large population in high need of acute medical care) and symptoms suggestive of SARS-CoV-2 infection is still a topical issue. The results of our study allow for the conclusion that, until receiving the result of a RT-PCR test for SARS-CoV-2 (usually 12–24 h), there are other markers useful for the differentiation of suspected patients with acute medical illnesses. Thus, the association with VTE as a comorbidity, fever, dry cough, and myalgias as clinical features, and GGO as a CT aspect are the main significant markers for the differentiation of patients with acute clinical syndromes suspected of having SARS-CoV-2. These data are useful for faster decision-making in the triage of COVID-19 patients before receiving RT-PCR test results, which can aid in avoiding keeping patients in crowded emergency departments. Our results can also aid doctors in not only relying only on biological markers for the detection of SARS-CoV-2 infection in patients with exacerbations of chronic diseases.

## Figures and Tables

**Table 1:** Case definition of severe acute respiratory syndrome coronavirus 2 (SARS-CoV-2) infection [7].

Any person with acute respiratory infection (sudden onset of at least one of the following symptoms: cough, fever, and dyspnea) OR Any person with severe acute respiratory infection (SARI) (fever or history of fever AND cough AND dyspnea AND need of hospitalization)

**Table 2:** Estimated risk of a positive SARS-CoV-2 RT-PCR test as a function of underlying chronic diseases.

Chronic Diseases	SARS-CoV-2 POS (*n* = 20)		SARS-CoV-2 NEG (*n* = 233)		*p*-Value for Chi^2^ Test	RR	95% CI
	*n*	%	*n*	%			
Obesity	5	25.0	51	21.9	0.751	1.17_POS_	0.45–3.09
Diabetes mellitus	4	20.0	50	21.5	0.878	1.01_NEG_	0.92–1.10
Hypertension	10	50.0	111	47.6	0.839	1.09_POS_	0.47–2.53
Chronic coronary syndrome	4	20.0	68	29.2	0.367	1.04_NEG_	0.96–1.11
Chronic heart failure	3	15.0	106	45.5	0.005	1.10_NEG_	1.03–1.18
Atrial fibrillation	1	5.0	64	27.5	0.011	1.10_NEG_	1.04–1.16
Venous thromboembolism	3	15.0	10	4.3	0.037	3.26_POS_	1.09–9.72
COPD	0	0.0	31	13.3	0.019	1.10_NEG_	1.05–1.15
Pulmonary fibrosis	3	15.0	43	18.5	0.694	1.02_NEG_	0.93–1.11
Liver cirrhosis	1	5.0	32	13.7	0.215	1.07_NEG_	0.99–1.14
Chronic kidney disease	2	10.0	56	24.0	0.120	1.06_NEG_	1.00–1.14
Bronchial asthma	1	5.0	6	2.6	0.564	1.85_POS_	0.29–11.9
Bronchiectasis	1	5.0	20	8.6	0.552	1.04_NEG_	0.94–1.15
Lung cancer	0	0.0	3	1.3	0.315	1.09_NEG_	1.05–1.13
Chronic hepatitis	3	15.0	36	15.5	0.957	1.01_NEG_	0.91–1.11
Inflammatory bowel disease	0	0.0	3	1.3	0.484	1.09_NEG_	1.05–1.13
Peptic ulcer	0	0.0	10	4.3	0.195	1.09_NEG_	1.05–1.13
Digestive cancer	0	0.0	12	5.2	0.099	1.09_NEG_	1.05–1.14

Chi^2^ test: chi-squared test; RR: relative risk; CI: confidence interval; POS: positive; NEG: negative; COPD: chronic obstructive pulmonary disease. A *p*-value of <0.05 was considered statistically significant.

**Table 3:** Clinical characteristics of SARS-CoV-2 POS and SARS-CoV-2 NEG patients.

Clinical Characteristic	SARS-CoV-2 POS (*n* = 20)		SARS-CoV-2 NEG (*n* = 233)		*p*-Value for Chi^2^ Test	Odds Ratio	95% CI
	*n*	%	*n*	%			
History of fever at home	12	60.0	74	31.8	0.013	3.22	1.26–8.22
Fever at presentation	5	25.0	43	18.5	0.488	1.47	0.51–4.27
Asthenia	7	35.0	64	27.5	0.481	1.42	0.54–3.32
Headache	4	20.0	24	10.3	0.222	2.18	0.67–7.05
Dry cough	12	60.0	79	33.9	0.023	2.92	1.15–7.45
Sputum production	3	15.0	34	14.6	0.961	1.03	0.29–3.72
Chest pain	6	30.0	47	20.2	0.320	1.70	0.62–4.65
Dyspnea	7	35.0	132	56.7	0.050	0.41	0.16–1.07
Myalgias	7	35.0	19	8.2	0.002	6.07	2.16–17.0
Dysphonia	0	0.0	7	3.0	0.279	-	-
Sore throat	3	15.0	9	3.9	0.025	4.39	1.09–17.8
Anosmia (loss of smell)	1	5.0	0	0.0	0.024	-	-
Dysgeusia (loss of taste)	1	5.0	1	0.4	0.113	12.2	0.74–203.0
Nausea	2	10.0	15	6.4	0.565	1.62	0.34–7.62
Vomiting	2	10.0	19	8.2	0.780	1.25	0.27–5.81
Abdominal pain	4	20.0	82	35.2	0.151	0.46	0.15–1.42
Diarrhea	4	20.0	30	12.9	0.395	1.69	0.53–5.40
Loss of appetite	3	15.0	10	4.3	0.037	3.94	0.99–15.7
Arthralgia	1	5.0	11	4.7	0.955	1.06	0.13–8.67

Chi^2^ test: chi-squared test; CI: confidence interval. A *p*-value of <0.05 was considered statistically significant.

**Table 4:** Evolution and hospitalization of SARS-CoV-2 POS and SARS-CoV-2 NEG patients. ICU: intensive care unit.

Evolution and Hospitalization	SARS-CoV-2 POS (*n* = 20)		SARS-CoV-2 NEG (*n* = 233)	
	*n*	%	*n*	%
Discharged improved	-	-	207	81.8
Transferred to Infectious Disease Hospital	20	7.9	-	-
Admitted to ICU	0	0.0	17	6.7
Deceased	0	0.0	26	10.3

**Table 5:** Characteristics of blood tests in SARS-CoV-2 POS and SARS-CoV-2 NEG patients. CRP: C-reactive protein; LDH: lactate dehydrogenase; CKMB: creatine kinase MB.

Characteristic	SARS-CoV-2 POS (*n* = 20)	SARS-CoV-2 NEG (*n* = 233)	*p*-Value (SARS-CoV-2 POS vs. SARS-CoV-2 NEG)
Decreased absolute value of lymphocyte count (˂1000/mm^3^), *N* (%)	12 (60.0%)	176 (75.5%)	0.121
Increased CRP (˃0.5 mg/dL), *N* (%)	15 (75.0%)	177 (76.0%)	0.814
Increased D-dimer (˃5 mcg/mL), *N* (%)	1 (5.0%)	35 (15.0%)	0.459
Increased LDH (˃214 IU/L), *N* (%)	7 (35.0%)	77 (33.0%)	0.893
Increased CKMB (˃25 IU/L), *N* (%)	3 (15.0%)	53 (22.8%)	0.709
Increased troponin (˃29 ng/L), *N* (%)	4 (20.0%)	56 (24.0%)	0.323
Increased ferritin (˃300 ng/mL), *N* (%)	3 (15.0%)	38 (16.3%)	0.130

A *p*-value of <0.05 was considered statistically significant.

**Table 6:** Estimated risks with regard to computed tomography (CT) findings as a function of SARS-CoV-2 RT-PCR test.

CT Changes	SARS-CoV-2 POS (*n* = 20)		SARS-CoV-2 NEG (*n* = 233)		*p*-Value for Chi^2^ Test	RR	95% CI
	*n*	%	*n*	%			
Ground-glass opacities	9	45.0	34	14.6	0.002	3.99_POS_	1.76–12.4
Consolidationaspects	1	5.0	33	14.2	0.197	1.06_NEG_	0.99–1.14
Ground-glass opacities + consolidation	5	25.0	57	24.5	0.957	1.03_POS_	0.92–1.09

**Table 7:** Univariate and multivariate logistic regression analyses for selected variables significantly associated with a positive SARS-CoV-2 RT-PCR test in patients with acute clinical syndromes.

Variables	Univariate Logistic Regression			Multivariate Logistic Regression		
	OR	95% CI	*p*-Value	OR	95% CI	*p*-Value
Venous thromboembolism	3.935	1.001–15.66	0.037	2.553	1.084–5.133	0.003
History of fever at home	3.223	1.264–8.219	0.013			
Dry cough	2.920	1.151–7.450	0.023			
Myalgia	6.072	2.162–17.02	0.002	1.886	1.744–5.727	0.005
Sore throat	4.390	1.091–17.80	0.025			
Loss of appetite	3.943	0.999–15.70	0.037	1.696	1.058–5.097	0.043
GGO on CT scan	4.320	1.617–11.54	0.002	1.671	1.640–4.749	0.005

OR: odds ratio; CI: confidence interval. A *p*-value of <0.05 was considered statistically significant.

**Table 8:** Diagnostic test performance as a function of clinical signs and CT appearance.

Variables	AUC	95% CI	*p*-Value	Sensitivity (%)	Specificity (%)
History of fever at home	0.641	0.512–0.771	0.036	65.0	62.0
Dry cough	0.630	0.501–0.760	0.050	65.0	60.0
Myalgia	0.634	0.491–0.778	0.046	75.0	45.0
Sore throat	0.556	0.415–0.697	0.409	55.0	52.0
Anosmia	0.525	0.388–0.662	0.711	52.0	50.0
Loss of appetite	0.554	0.412–0.694	0.427	55.0	51.0
GGO on CT scan	0.652	0.513–0.791	0.024	85.0	45.0

AUC: area under the curve; CI: confidence interval. A *p*-value of <0.05 was considered statistically significant.

## Data Availability

The data presented in this study are available on request from the corresponding author.
